# The Dutch Y-chromosomal landscape

**DOI:** 10.1038/s41431-019-0496-0

**Published:** 2019-09-05

**Authors:** Eveline Altena, Risha Smeding, Kristiaan J. van der Gaag, Maarten H. D. Larmuseau, Ronny Decorte, Oscar Lao, Manfred Kayser, Thirsa Kraaijenbrink, Peter de Knijff

**Affiliations:** 1 Department of Human Genetics, Leiden University Medical Center, Leiden, The Netherlands; 2 Forensic Biomedical Sciences, Department of Imaging & Pathology, KU Leuven, Leuven Belgium; 3 Laboratory of Socioecology and Social Evolution, Department of Biology, KU Leuven, Leuven Belgium; 4 Histories vzw, Mechelen, Belgium; 5 Laboratory of Forensic Genetics and Molecular Archaeology, UZ Leuven, Leuven Belgium; 6 Department of Genetic Identification, Erasmus MC University Medical Center Rotterdam, Rotterdam, The Netherlands; 7 Present Address: Department of Human Biological Traces, Netherlands Forensic Institute, The Hague, The Netherlands; 8 Universitat Pompeu Fabra, Barcelona, Spain; 9 Present Address: CNAG-CRG, Centre for Genomic Regulation (CRG), The Barcelona Institute of Science and Technology, Barcelona, Spain

**Keywords:** Population genetics, Genetics research

## Abstract

Previous studies indicated existing, albeit limited, genetic-geographic population substructure in the Dutch population based on genome-wide data and a lack of this for mitochondrial SNP based data. Despite the aforementioned studies, Y-chromosomal SNP data from the Netherlands remain scarce and do not cover the territory of the Netherlands well enough to allow a reliable investigation of genetic-geographic population substructure. Here we provide the first substantial dataset of detailed spatial Y-chromosomal haplogroup information in 2085 males collected across the Netherlands and supplemented with previously published data from northern Belgium. We found Y-chromosomal evidence for genetic–geographic population substructure, and several Y-haplogroups demonstrating significant clinal frequency distributions in different directions. By means of prediction surface maps we could visualize (complex) distribution patterns of individual Y-haplogroups in detail. These results highlight the value of a micro-geographic approach and are of great use for forensic and epidemiological investigations and our understanding of the Dutch population history. Moreover, the previously noted absence of genetic-geographic population substructure in the Netherlands based on mitochondrial DNA in contrast to our Y-chromosome results, hints at different population histories for women and men in the Netherlands.

## Introduction

An extensive database of genetic variation and detailed insight in genetic-geographic population substructure is essential for forensic investigation, epidemiology and historical, archaeological, evolutionary and genealogy studies within a nation (see for example [1]). For the Netherlands there are several databases available on autosomal data from which inferences have been made about Dutch population (sub)structure. The study by Lao et al. [2] on genome-wide autosomal single nucleotide polymorphism (SNP) data demonstrated genetic-geographic population substructure and a clinal distribution of genomic diversity in southeast to northwest direction across the current territory of the Netherlands. They concluded that these patterns must have a relatively recent origin, considering multiple recent events that could have influenced the Dutch population structure, such as large-scale land reclamation projects starting in the High Middle Ages. Furthermore, Abdellaoui et al. [3], using an independent genome-wide dataset, observed population differentiation along both a north–south and a west-east direction and identified a higher rate of homozygosity in the north compared with the south, which was explained by a serial founder effect as a result of historical northward migrations. Similarly, also the study of The Genome of the Netherlands Consortium [4] on whole-genome sequencing data observed subtle genetic-geographic substructure along a north–south gradient and also increased homozygosity in the north, for which they proposed the same explanation as Abdellaoui et al. [3]. In contrast to the autosomal genome-wide data, a study on mitochondrial DNA on a subset of the above mentioned dataset studied by Lao et al. [2], could not detect significant genetic-geographic substructure in the Netherlands [5].

Considering the non-recombining part of the Y-chromosome, Roewer et al. described a significant division between the north and the south of the Netherlands, based on Y-chromosomal short tandem repeat (YSTR) haplotypes from 275 samples from five different locations, but this division was not detectable based on SNP data [6]. Moreover, studies on SNP based Y-chromosome haplogroup (YHG) frequencies in the southern Netherlands and Belgium showed a significant difference between the Dutch and the Belgian samples based on haplogroup proportions and gradients for YHGs R1b-M405, R1b-L48, and R1b-M529 [7–9].

Despite the aforementioned studies, YHG data from the Netherlands remain scarce and do not cover the territory of the Netherlands well enough to allow a reliable investigation of genetic-geographic population substructure. Such a Dutch YHG database would be a valuable addition to the already available autosomal and mitochondrial DNA information for various reasons. First of all, in contrast to the autosomes the Y-chromosome is uniparental. This means its genetic variation is influenced by, but also indicative of, male-specific demographic processes and is therefore very useful in reconstructing population histories. Also, in the forensic world genome wide data analysis is still scarcely applied (and standardized) and therefore uniparental markers remain essential for inferring bio-geographic ancestry of suspects or unidentified victims. Therefore, regional knowledge on genetic-geographic substructure of the Y-chromosome is of great value.

The phylogenetic resolution of the current YHG tree is now sufficiently high [10] to be able to detect geographic patterns on a micro-regional scale [7]. In this context, the Netherlands can be considered a micro-region, covering only 33,687 km^2^ of land [11], although it is densely populated with about 17 million inhabitants [12]. The goal of this study is to develop a database with Dutch YHG information and identify and quantify the possible presence of geographic patterns and population substructure based on this data within the Netherlands. For some of our analyses, we also included data from the northern part of Belgium, including Flanders and the Brussels-Capital region. This part of Belgium borders the south of the Netherlands and Dutch is (one of) the official language(s) there. This area covers 13,684 km^2^ of land [13, 14] and has about 7.5 million inhabitants [15].

## Materials and methods

**Fig. 1:**
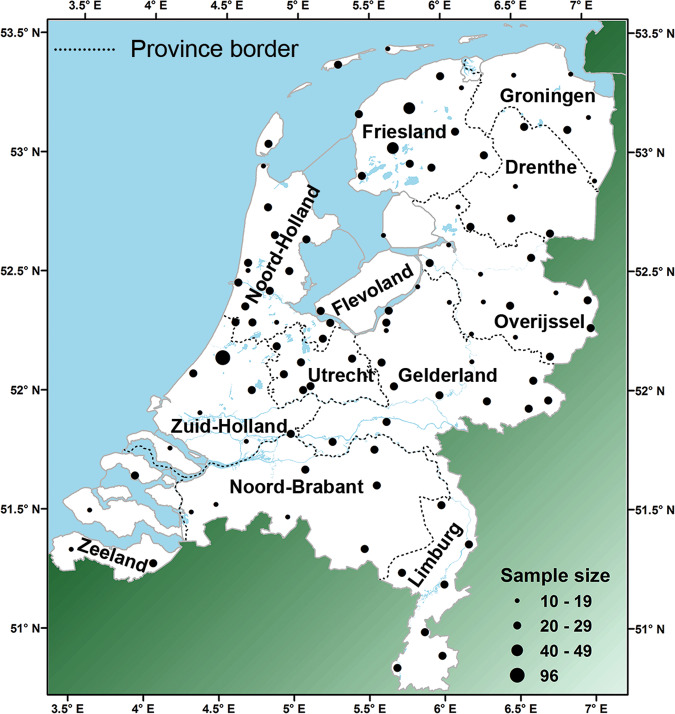
Map of the Netherlands with sample locations and province names

For our study, we used 2085 blood-donor samples from male donors that are reported to be unrelated and residing in a-priori selected locations. This is the same dataset as published in Westen et al. for autosomal STR data [16] and Westen et al. for YSTR data [17]. Samples were received anonymously, with only the place of residency of the donor indicated (see also Supplementary information). The number of samples per location varied from 1 to 96. Locations with less than ten samples were pooled with nearby locations. This resulted in sample sizes varying between 10 and 96 (average = 21) from 99 locations covering the Dutch area in a grid-like scheme (Fig. 1 and Table S1). This dataset will from here on be referred to as the “Dutch dataset”.

**Fig. 2:**
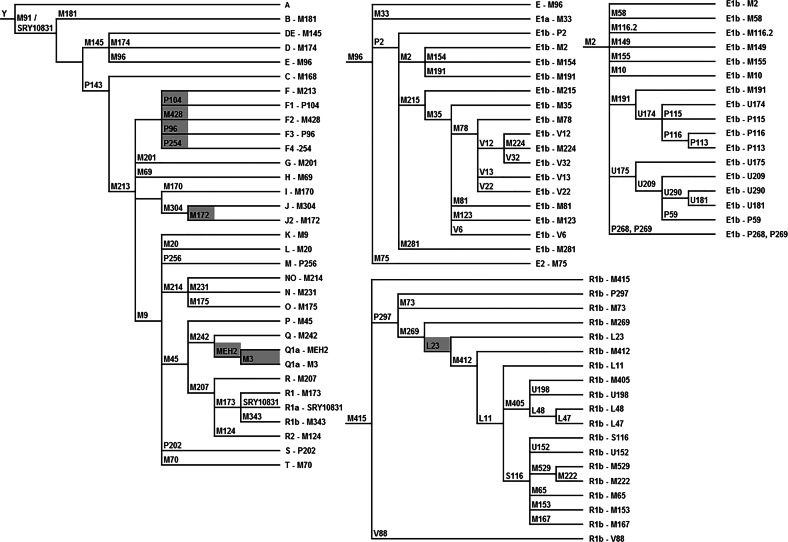
Typed SNPs in phylogenetic trees, grouped in the core set and the E-M96, E1b-M2, and R1b-M415 multiplex assays. Monoplexes are marked gray. SNP P143 in the core set multiplex was not typed but recommended instead of M168

Because of the sampling strategy, excluding several of the major cities that harbor large recent (since 1950) immigrant populations [18], this dataset is to some extend biased in representing the full genetic diversity among the Dutch male population. DNA was isolated as described in Westen et al. [16]. A SNaPshot® Multiplex System Kit (Applied Biosystems, Foster City, CA, USA) assay was designed for a core set of 26 SNPs covering all the main YHGs (A-T) and four subgroups of YHG R (Fig. 2). Further subtyping of samples assigned to YHGs E and R1b was done in additional multiplexes and for YHGs F(xG, H, I/ J, and K), J, and Q in monoplex (Fig. 2). In total, 92 Y-SNPs were analyzed allowing the inference of 88 YHGs. Subgroups of YHG I-M170 were inferred with Whit Athey’s Haplogroup Predictor [19, 20], based on 16 YSTRs that were previously published for our dataset [17]. Because sub-haplogroups of I-M170 were inferred from YSTR data, they were not considered for further analysis, other than proportion estimates, due to potential inference inaccuracies. For detailed information on SNP design, sequencing, and haplogroup prediction see Supplementary information.

To increase the study area and enhance the possibility to detect geographic patterns, a dataset of 773 males from the northern Belgian provinces West-Vlaanderen, Oost-Vlaanderen, Antwerpen, Vlaams-Brabant, the Brussels region, and Limburg, using the actual living place at the time of sample collection (the “present” dataset), as previously described by Larmuseau et al. [7], was incorporated in this study for part of the statistical analyses. This dataset will be referred to as the “Flanders dataset” and the dataset consisting of both the Dutch and the Flanders dataset will be referred to as the “combined dataset”. Because there were some differences between the SNP assays applied to the Dutch and Flanders datasets, YHGs were synchronized to a level that they were comparable for data analysis (consensus YHGs, Table S2).

The samples from the Flanders dataset were collected with a different sampling strategy than the Dutch dataset and mostly single samples are available per location. Therefore, several methods for detecting population substructure and gradients were not applicable to the Flanders dataset.

We used two different approaches in order to search for genetic-geographic patterns in the Dutch dataset. First, classical multidimensional scaling (MDS) analysis was performed with R software version 3.4.4 [21] to represent in two dimensions a distance matrix based on Slatkin’s linearized *F*_ST_ on YHG proportions among sampling locations. This matrix was computed with ARLEQUIN software version 3.11 with standard settings [22]. The resemblance of the first two MDS dimensions with the geographic sampling locations was quantified by means of a symmetric Procrustes rotation, as implemented in the protest method of the “vegan” R package [23]. Second, a correspondence analysis on YHG proportions and sample locations was performed in R with the “ca” package. Outliers, if any, in the first two dimensions were removed on visual inspection. The first two dimensions were compared with the geographic coordinates of the sampling locations with a symmetric Procrustes rotation. Both methods were only applied to the Dutch dataset.

The above mentioned previous study by Lao et al. [2] on genome-wide autosomal SNP data was based on a subset of the Dutch dataset as published here. This study found evidence for genetic-geographic substructure in the Dutch population. To test for similarities between genetic-geographic substructure based on the genome-wide autosomal SNP data and YHG data we estimated the similarity between the Slatkin’s linearized *F*_ST_ genetic distance matrices between populations while controlling by geographic distance, by conducting a partial Mantel test with PASSaGE version 2 software [24]. Since the dataset of Lao et al. [2] is a subset of the Dutch dataset (917 samples from 46 locations), we applied the same selection on the YHG dataset (applying all selection criteria as described in Lao et al. [2] and additionally excluding all locations with less than ten samples).

To detect geographic patterns for individual YHGs, we applied two statistical methods on (sub)YHGs with a proportion of ≥1% (corresponding to a minimum of 20 samples in the Dutch dataset). First, we computed Moran’s I spatial autocorrelograms with binary weight tests for each YHG using PASSaGE version 2 software, with ten distance classes of equal width, assuming randomly distributed data and excluding the largest distance class. Statistical significance (*p*-value < 0.05) of the spatial autocorrelogram of each YHG was estimated with 999 permutations and Bonferroni correction was applied. This test was only applied to the Dutch dataset. Second, we estimated the linear relationship between the proportion of each YHG and geographic coordinates with a generalized linear model (GLM) using a binomial logit link in R with the “vegan” package. Bonferroni correction was applied on the resulting *p*-values for the correlation (0.05/(number of tested YHGs (excluding R1b-M405 Total and R1b-S116 Total) +1 for not tested haplogroups)). Gradients that are significant before Bonferroni correction, but no longer after, will be referred to as marginally significant. This test was applied to both the Dutch and the combined dataset.

To visualize spatial patterns, we created prediction surface maps for all YHGs with a proportion of ≥1% with the ordinary Kriging interpolation technique in ArcGIS version 10.2 with the Spatial Analyst extension. Data were classified in equal intervals, unless the distribution of the data required otherwise. The number of classes was defined manually, depending on the range of predicted values. This was only done for the Dutch dataset.

Because YHGs R1b-M405 and R1b-S116 were often not or to a limited extend subtyped in previous publications, we also provide collective information for these YHGs under the names of R1b-M405 Total (comprising M405, U198, L48, and L47) and R1b-S116 Total (comprising S116, U152, M529, and M167).

Data generated in this study have been uploaded to the public database YHRD under accession number YA002897 [25, 26].

## Results

**Fig. 3:**
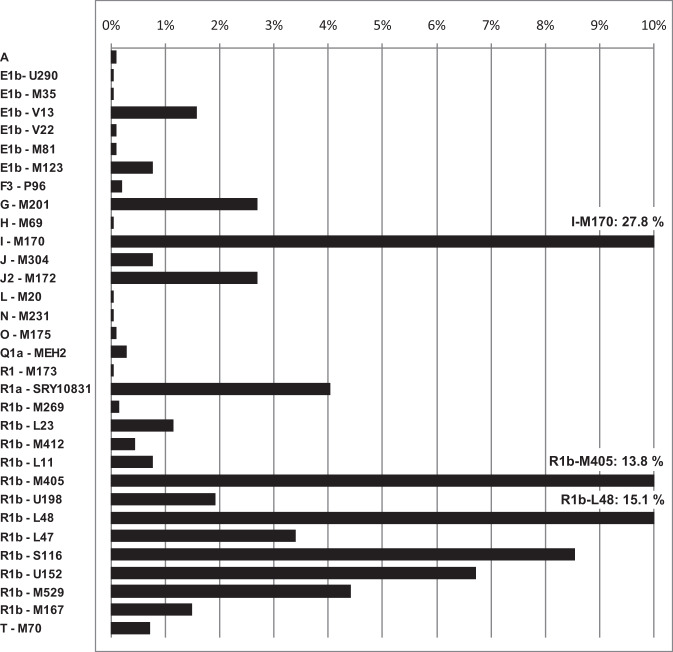
Graph of YHG proportions in the Dutch dataset

YHGs were manually assigned to all 2085 samples of the Dutch dataset. In total, 32 different YHGs were observed. YHGs I (28%) and R (62%) are by far the most common. Within YHG R, subgroups R1b-L48 (15%), R1b-M405 (14%), and R1b-S116 (9%) are the three most common ones (Table 1, Fig. 3). The combined dataset also mainly contains YHGs I (26%) and R (63%) (Table 2).

**Table 1:** YHG proportions in the Dutch dataset

YHG	#	%
A	2	0.10
**E Total**	**55**	**2.64**
E1b—U290	1	0.05
E1b—M35	1	0.05
E1b—V13	33	1.58
E1b—V22	2	0.10
E1b—M81	2	0.10
E1b—M123	16	0.77
F3—P96	4	0.19
G—M201	56	2.69
H—M69	1	0.05
I—M170	580	27.82
**J Total**	**72**	**3.45**
J—M304	16	0.77
J2—M172	56	2.69
L—M20	1	0.05
N—M231	1	0.05
O—M175	2	0.10
Q1a—MEH2	6	0.29
**R Total**	**1292**	**61.97**
R1—M173	1	0.05
R1a—SRY10831	84	4.03
R1b—M269	3	0.14
R1b—L23	24	1.15
R1b—M412	9	0.43
R1b—L11	16	0.77
**R1b—M405 Total**	**712**	**34.15**
R1b—M405	287	13.76
R1b—U198	40	1.92
R1b—L48	314	15.06
R1b—L47	71	3.41
**R1b—S116 Total**	**441**	**21.15**
R1b—S116	178	8.54
R1b—U152	140	6.71
R1b—M529	92	4.41
R1b—M167	31	1.49
T—M70	15	0.72
**Total**	**2085**

**Table 2:** YHG proportions in the Combined, Flanders and Dutch datasets based on consensus YHGs

Consensus YHG	Combined dataset		Flanders dataset		Dutch dataset	
	#	%	#	%	#	%
A	3	0.10	1	0.13	2	0.10
**E Total**	94	**3.29**	**39**	**5.05**	**55**	**2.64**
E1b—U290	1	0.03	0	0.00	1	0.05
E1b—M215	2	0.07	2	0.26	0	0.00
E1b—M35	1	0.03	0	0.00	1	0.05
E1b—M78	1	0.03	1	0.13	0	0.00
E1b—V12	1	0.03	1	0.13	0	0.00
E1b—V13	55	1.92	22	2.85	33	1.58
E1b—V22	5	0.17	3	0.39	2	0.10
E1b—M81	4	0.14	2	0.26	2	0.10
E1b—M123	24	0.84	8	1.03	16	0.77
F3—P96	4	0.14	0	0.00	4	0.19
G—M201	84	2.94	28	3.62	56	2.69
H—M69	1	0.03	0	0.00	1	0.05
I—M170	737	25.79	157	20.31	580	27.82
**J Total**	**110**	**3.85**	**38**	**4.92**	**72**	**3.45**
J—M304 (xM172)	25	0.87	9	1.16	16	0.77
J2—M172	85	2.97	29	3.75	56	2.69
L—M20	4	0.14	3	0.39	1	0.05
N—M231	1	0.03	0	0.00	1	0.05
O—M175	2	0.07	0	0.00	2	0.10
Q1—P36.2	9	0.31	3	0.39	6	0.29
**R Total**	**1791**	**62.67**	**501**	**64.81**	**1290**	**61.87**
R1—M173	2	0.07	1	0.13	1	0.05
R1a—SRY10831	110	3.85	26	3.36	84	4.03
R1b—M343	1	0.03	1	0.13	0	0.00
R1b—P297	1	0.03	1	0.13	0	0.00
R1b—M269	76	2.66	24	3.10	52	2.49
**R1b—M405 Total**	**906**	**31.70**	**194**	**25.10**	**712**	**34.15**
R1b—M405	381	13.33	94	12.16	287	13.76
R1b—U198	49	1.71	9	1.16	40	1.92
R1b—L48	476	16.66	91	11.77	385	18.47
**R1b—S116 Total**	**695**	**24.32**	**254**	**32.86**	**441**	**21.15**
R1b—S116	284	9.94	106	13.71	178	8.54
R1b—U152	221	7.73	81	10.48	140	6.71
R1b—M529	151	5.28	59	7.63	92	4.41
R1b—M167	39	1.36	8	1.03	31	1.49
T	18	0.63	3	0.39	15	0.72
**Total**	**2858**		**773**		**2085**	

**Fig. 4:**
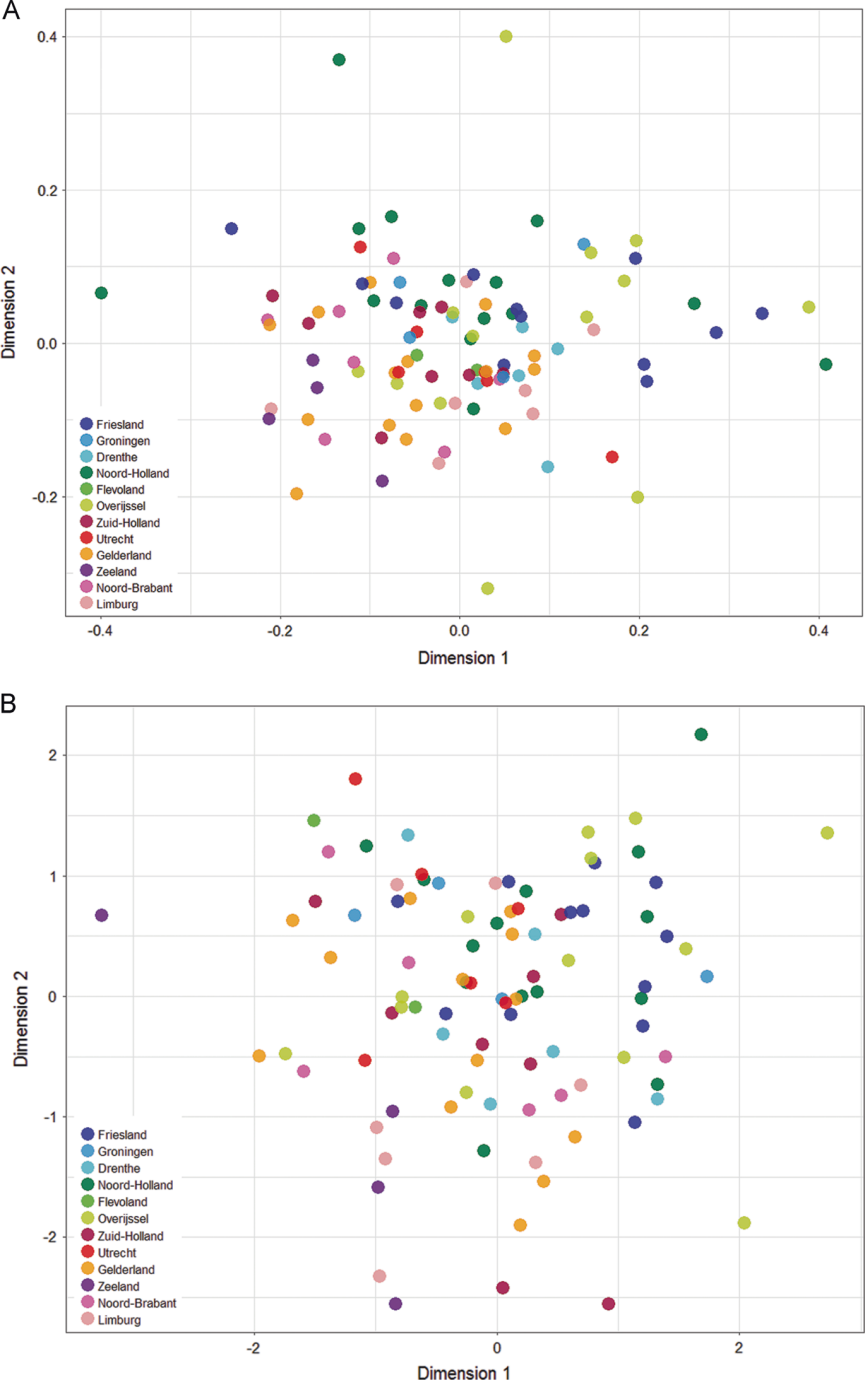
**a** Plot of the first two dimensions from the classical multidimensional scaling analysis. **b** Plot of the first two dimensions from the correspondence analysis without outliers. Sample locations are colored by province

The first two dimensions of the MDS analysis using a genetic distance matrix between populations explained 4.8% and 2.8% of the total YHG proportion variance, respectively. There is a statistically significant, weak positive correlation between these two dimensions and the geographical coordinates of the sampling locations as estimated by a Procrustes analysis (*r* value = 0.37, *p*-value = 0.001 after 999 permutations) (Fig. 4a).

The first two dimensions of the correspondence analysis using a sample by YHG frequency contingency table explain 8.8% and 7.9% of the total variance, respectively. There is no statistically significant correlation between these two dimensions and the geographical coordinates of the sampling locations with a Procrustes rotation (Fig. S1). Visual inspection of the correspondence analysis plot, however, showed several putative outliers (the locations Harderwijk, Purmerend, Putten and Veghel) (Fig. S1). When these locations are excluded from the analysis, the first two dimensions of the correspondence analysis explain 8.6% and 7.4% of the total variance. This time there is a statistically significant, weak positive correlation between these two dimensions and the geographical coordinates of the sampling locations with a Procrustes rotation (*r* value = 0.268, *p*-value = 0.002 after 999 permutations) (Fig. 4b).

When comparing the new YHG data with the previous genome-wide autosomal SNP data from Lao et al. [2], we did not observe a statistically significant correlation between the genetic distance matrices based on Slatkin’s *F*_ST_ between the two datasets after controlling by geographic distance (*r* = 0.005, 2-tailed *p*-value partial Mantel test = 0.954 after 999 permutations).

The spatial analyses of individual YHGs identified departure from spatial randomness for several of them. The spatial autocorrelograms suggest statistically significant spatial trends compatible with a cline [27] after Bonferroni correction for the following YHGs in the Dutch dataset: G-M201, I-M170, R1b-M405, R1b-S116 Total, and R1b-S116 (Table 3, Fig. S2). The GLM results for the Dutch dataset also indicate (marginally) significant correlation with latitude and/or longitude for several YHGs, where G-M201, R1b-L23, R1b-S116 Total, R1b-S116, and R1b-U152 increase from north to south, R1b-M405 Total and R1b-L48 increase from south to north, R1b-M405 increases from east to west and I-M170 increases from southwest to northeast (Table 3). In the combined dataset, the GLM also indicates (marginally) significant correlation with latitude and/or longitude for several YHGs, where YHGs G-M201, J2-M172, R1b-M269, and R1b-S116 increase from north to south, R1b-M405 Total and R1b-L48 increase from south to north, I-M170 increases from southwest to northeast and R1b-S116 Total, R1b-U152 and R1b-M529 increase from northeast to southwest (Table 4).

**Table 3:** Results of the GLM and Moran’s I spatial autocorrelograms for the Dutch dataset

YHG ≥ 1 %	Generalized linear model					Moran’s I spatial autocorrelograms	
	Direction of increase	Latitude		Longitude			
		Slope	*p*-value	Slope	*p*-value	*p*-value	Pattern
E1b—V13		0.379	0.2150	0.216	0.3150	0.0660	
**G—M201**	S	**−0.735**	**0.0011**	0.118	0.4712	**0.0005**	Cline
**I—M170**	NE	**0.262**	**0.0018**	**0.222**	**0.0002**	**0.0009**	Cline
J2—M172		**−**0.380	0.0930	**−**0.189	0.2455	0.7649	
R1a—SRY10831		**−**0.145	0.4403	0.029	0.8312	0.2812	
**R1b—L23**	S	**−***0.862*	*0.0116*	**−**0.051	0.8369	0.1721	
**R1b—M405 Total**	N	**0.302**	**0.0001**	**−**0.086	0.1216	**0.0096**	Cline
**R1b—M405**	W	0.078	0.4725	**−***0.169*	*0.0271*	0.8904	
R1b—U198		0.189	0.4898	**−**0.040	0.8332	1.0000	
**R1b—L48**	N	**0.405**	**0.0001**	0.022	0.7656	0.1582	
R1b—L47		0.117	0.5697	**−**0.040	0.7847	0.1690	
**R1b—S116 Total**	S	**−0.366**	**0.0001**	**−**0.051	0.4257	**0.0073**	Cline
**R1b—S116**	S	**−0.486**	**0.0002**	**−**0.042	0.6589	**0.0004**	Cline
**R1b—U152**	S	**−***0.318*	*0.0300*	**−**0.124	0.2383	1	
R1b—M529		**−**0.195	0.2750	0.060	0.6416	1	
R1b—M167		0.400	0.2054	**−**0.004	0.9855	1	

For the GLM results: negative values for the slope indicate increase from north to south (Latitude) or east to west (Longitude), and positive values indicate a reverse direction of increase; significant *p*-values before Bonferroni correction (*p*-value of 0.0033) are shown in italic, and after Bonferroni correction in bold. For the spatial autocorrelograms: *p*-values are Bonferroni corrected. All YHGs with (marginally) significant *p*-values are shown in bold

**Table 4:** Results of the GLM for the combined dataset

Consensus YHG ≥1 %	Direction of increase	Latitude		Longitude	
		Slope	*p*-value	Slope	*p*-value
E1b—V13		**−**0.211	0.2478	0.031	0.8269
**G—M201**	S	**−0.458**	**0.0029**	0.028	0.8097
**I—M170**	NE	**0.288**	**0.0000**	**0.208**	**0.0000**
J2—M721	S	**−***0.304*	*0.0420*	**−**0.209	0.0698
R1a—SRY10831		0.020	0.8752	0.055	0.5888
**R1b—M269**	S	**−***0.440*	*0.0062*	**−**0.226	0.0626
**R1b—M405 Total**	N	**0.323**	**0.0000**	0.047	0.2697
R1b—M405		0.093	0.2046	**−**0.044	0.4429
R1b—U198		0.233	0.2252	0.083	0.5833
**R1b—L48**	N	**0.399**	**0.0000**	0.100	0.0592
**R1b—S116 Total**	SW	**−0.427**	**0.0000**	**−0.167**	**0.0003**
**R1b—S116**	S	**−0.452**	**0.0000**	**−**0.123	0.0595
**R1b—U152**	SW	**−0.359**	**0.0002**	**−***0.155*	*0.0346*
**R1b—M529**	SW	**−0.345**	**0.0024**	**−***0.198*	*0.0237*
R1b—M167		0.331	0.1256	0.105	0.5371

Negative slope values indicate increase from north to south (latitude) or east to west (longitude); positive values indicate a reverse direction of increase; significant *p*-values before Bonferroni correction (*p*-value of 0.0033) are shown in italic, and after Bonferroni correction in bold. All YHGs with (marginally) significant *p*-values are shown in bold

**Fig. 5:**
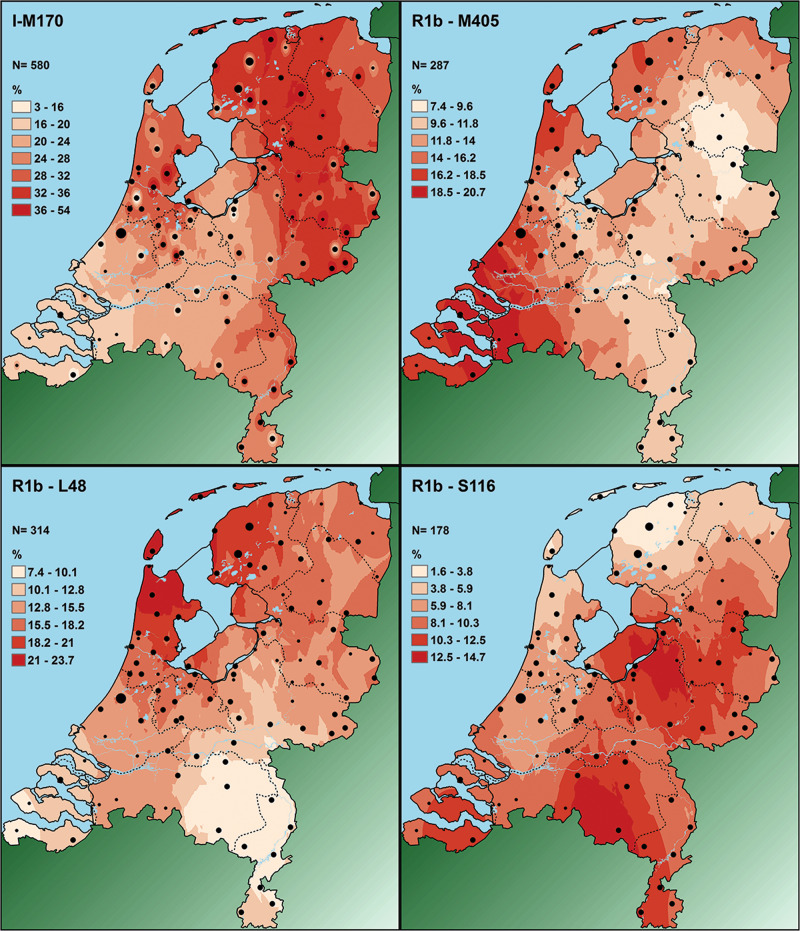
Prediction surface maps of the four most frequent (sub-)YHGs in the Dutch dataset in phylogenetic order

From all the YHGs for which prediction surface maps were created, only YHG R1b-M529 is more or less evenly distributed over the Netherlands. All other YHGs show more distinct patterns of distribution, either clinal or non-clinal and more patchy (Figs. 5 and S3). The prediction surface maps in general support the statistically (marginally) significant patterns found with the spatial autocorrelograms and/or GLM analyses, but also allow for a more elaborate description of the pattern of distribution. In accordance to the spatial autocorrelogram and GLM result, the prediction surface map of YHG G-M201 shows a southward increase in proportion, although the overall frequency is low. However, the observed pattern appeared more complex than just a cline, best described as an inverse saddle pattern. The prediction surface map of YHG I-M170 is the only one for which we did not classify the frequency in equal intervals. Overall, the range of frequency per location was very large compared with the other YHGs, running from 3% to 54%. There were, however, hardly any observations below 16% and above 36%. We therefore partly applied classification by natural breaks, where the lowest classes, between 3% and 16% were pooled in one class and the highest classes, between 36% and 54%, were pooled in one class. Between 16% and 36% we applied equal intervals. What stands out most for this map, in comparison to the others, are the distinct local higher or lower proportions compared with the surrounding area, despite the clearly visible increase from southwest to northeast. What is striking about the distribution of YHG R1b-L23, is that the proportions are very low for the whole country, apart from a limited number of local concentrations of somewhat higher proportions. Although proportions are slightly higher in the south, the map does not really support the marginally significant southward gradient from the GLM model. The map for YHG R1b-M405 Total suggests a gradient from southeast to northwest, while with the GLM for R1b-405 Total only a significant northward gradient was found.

The map for YHG R1b-M405 shows a gradient that runs from the northeast to the southwest, while only a marginally significant westward gradient was observed with the GLM. For YHG R1b-L48 the map suggests a gradient in a northwestern direction, rather than the significant northward gradient as indicated by the GLM results. For YHGs R1b-S116 Total, R1b-S116, and R1b-U152, the maps show similar patterns and suggest more southeastwards gradients, rather than the southward gradients as suggested by the GLM results.

Furthermore, the prediction surface maps suggest the presence of other complex patterns that could not be identified with the statistical analyses we performed, or were not significant, mostly due to low proportions. For YHG E1b-V13, we observe a depression-like pattern with highest proportions in the southwest and northeast and a band of lower proportions running from the northwest to the southeast. YHG J2-M172 shows a complex pattern with dispersed patches of higher and lower proportions. For R1a-SRY10831 we see two distinct areas of higher concentrations in the center and northeast of the country. YHG R1b-U198 seems to follow a depression-like pattern, somewhat similar to E1b-V13, with highest proportions in the southwest and northeast and a band of lowest proportioning the center. We see a more or less random distribution for R1b-L47, except for a distinct vertical band of low proportions in the northern half of the country and also rather distinct patches of high proportions in the northeast and center. Finally, the map for R1b-M167 indicates a clear concentration of low proportions in the southeast and increasing proportions towards the northwest.

## Discussion

The Y-chromosomal genetic diversity present in the Dutch dataset showed clear spatial differentiation. What is remarkable, considering the small size of the Netherlands, is that additionally we see a non-random spatial pattern, either by formal testing or visually in the prediction surface maps, for nearly every YHG tested (≥1%). Roewer et al. already detected population substructure in the form of a division between two samples from the south of the Netherlands and three samples from the central-west and north. This was based, however, on YSTR data and could not be replicated with YSNP data from a smaller selection of that same sample set [6]. The contrast between the division they detected with the YSTR data and the more gradual patterns we observed here based on YSNP data are probably related to the relatively small sample set and geographically far dispersed sample locations in Roewer et al., but may also be related to fundamental differences between YSTRs and YSNPs, such as mutation rate. The contrast between the lack of substructure based on YSNP data in Roewer et al. and our results are probably related to the low resolution of YSNP typing (only those to assign to the most common European YHGs) and also the small sample set in the study of Roewer et al. This also demonstrates the value of a substantial and geographically well distributed dataset with detailed YSNP information.

The population substructure and gradients for many of the individual YHGs we found in our study are in strong contrast with the apparent lack of genetic-geographic patterns for mtDNA data, as previously reported for a subset of the same sample set we used for our study [5]. This could be an indication of different demographic histories for women and men. One could think, for example, of the patrilocal residence system, which is typical for farming societies, such as the Dutch [28]. In these societies sons stay with their family and daughters move to the residence of their husbands. Also, genetic drift may have acted differently on mt-DNA than on Y-chromosomes.

The dissimilarity between the geographic-genetic patterns we observed for the Y-chromosome and the one found for genome-wide autosomal SNP data by Lao et al. [2], based on a selection of our dataset, can first of all most likely be attributed to genetic drift acting differently on autosomal DNA than on uniparental markers such as the Y-chromosome. Besides that, the autosomal genetic variation and distribution are affected by both female and male populations and therefore the gradients we observed for the male population by means of the YHG data will be diluted by the lack of gradients for the female population by means of the mtSNP data.

A solid explanation, however, for the differences in the geographic-genetic patterns between autosomal, mitochondrial, and Y-chromosomal SNP data requires additional data from past periods by means of ancient DNA and multidisciplinary research, including historians and archaeologists. Larmuseau et al. already hinted at the value of temporal analyses with several studies where they reconstructed historical patterns based on a combination of present-day samples and genealogical data and found strong indications for temporal fluctuations of YHG proportions [7, 8].

When we additionally include the Flanders dataset in our analyses, we detect almost the same gradients with formal testing. This is not surprising since all but one of the significant gradients we observed in the Dutch dataset run in a (more or less) longitudinal direction and the inclusion of the Flanders dataset extends the territory mostly southwards. However, two exceptions concern YHGs R1b-M405 and R1b-M529. For R1b-M405, we detected a marginally significant westward gradient in the Dutch dataset, but no significant gradient in the combined dataset. This is a remarkable observation, since it is one of the three main subgroups in both the Dutch and Flanders datasets. For R1b-M529 we did not detect a significant gradient in the Dutch dataset, while we do detect a (marginally) significant south(west)ward gradient in the combined dataset.

All together our results are in line with the findings of Larmuseau et al. [8] of significant population differentiation between the provinces of the historical region of the Duchy of Brabant, currently covering the Dutch province of Noord-Brabant and the Belgian provinces of Antwerpen, Brussel, Vlaams Brabant, and Waals Brabant. This was interpreted as the result of isolation by distance, rather than a distinct difference between the present-day Dutch and Belgians. Considering the many north–south clines we observed we can support this assumption.

There are two other comparable examples of YSNP based population studies in the vicinity of the Netherlands; one for Germany and Poland by Kayser et al. [29] and one for France by Ramos-Luis et al. [30], although both subtyped to a considerably lower resolution than we did. Kayser et al. detected a significant west to east gradient for YHG R1a1 and a reverse gradient for YHG R1(xR1a1) across Germany and Poland. Within Germany, they found Y-chromosome differentiation between Western Germany and Eastern Germany, which the authors explained by more ancient events such as stronger Slavic influence on eastern than on western parts of Germany. In our study we did not subtype further than R1a, but this YHG is not very common in our dataset  (4%) and shows no gradient. Overall though, it’s frequency fits the eastward increasing gradient as detected by Kayser et al. Since R1(xR1a1) was not further subtyped and we detected contradictory gradients within R1b, it is not possible to compare patterns.

In the French study by Ramos-Louis et al. on samples from seven regions they reported no clear indications for population substructure, other than Bretagne being a genetic outlier [30]. This in itself is remarkable for such a large country. Considering their resolution of SNP typing we could compare their data with our significant gradients for YHGs G, I, and R1b-M405 Total, but in neither case do we see evidence for the continuation of the gradients we observed.

To further put our results in a broader context, we compare the (marginally) significant YHG gradients in the Dutch dataset with published geographic distribution patterns in Europe. According to Rootsi et al. [31] YHG G-M201 is relatively rare in Europe, with average proportions below 5% in northwestern Europe, which is consistent with our findings. Because proportions are low throughout the most of Europe, there is no clear gradient, but overall it increases from northwest to southeast, similar to the gradient observed in the Dutch dataset with the GLM and prediction surface map. The geographical distribution of YHG I-M170 in Europe, on the other hand, is more complex, with two centers of high concentrations: one in Scandinavia and one in southern Europe around the Dinaric Alps [32]. In northwest Europe, though, it shows an increase from southwest to northeast, which is similar to the trend we observed in the Dutch dataset. YHG R1b-L23 was studied in Myres et al. [33], but since this YHG is so rare in Western Europe, as we also see in the Dutch dataset, genetic-geographic patterns are absent for Europe. For R1b-M405 and R1b-L48 no other European-wide data are available, and we are therefore limited to compare just R1b-M405 Total. In both Myres et al. [33] and Busby et al. [34] R1b-M405 Total is depicted with a more or less concentric distribution, with the Netherlands as the center. It is therefore difficult to compare the European trend with the Dutch one, which increases from southeast to northwest, although statistically only the north–south cline is significant. The proportions that they observed are similar to our observations, however. According to Myres et al. R1b-S116 shows a southwestern to northeastern decrease in Europe, while in the Dutch dataset the gradient is directed from northwest to southeast. Average proportions are more or less similar though [33]. R1b-U152 has relatively high proportions in central–southern Europe and lower proportions on the peripheral areas. The gradient and proportions as observed in the Dutch dataset are similar to those described in Myres et al. [33].

The (marginally) significant trends that are observed in our study in the Dutch dataset in most cases seem to more or less follow the European-wide trends for YHG distributions. The fact that our results resemble these European trends could be interpreted as the result of European-wide events. However, recent studies on both modern [35, 36] and ancient populations [37–40] indicate the importance of including data from past populations when trying to explain the present picture. Others also argue that local, but also more recent, demographic events may have had just as much or even more influence on the distribution of current genetic variation and produce similar patterns as major events in prehistory [41–43].

In summary, our detailed analysis of YHG distribution in the Netherlands, based on 2085 geographically dispersed males resulted in an informative database. By combining several statistical methods, we have been able to detect Y-chromosome based genetic-geographic population substructure and significant gradients for several individual YHGs in the Netherlands, some of which extend to the south into the northern part of Belgium and eastward into Germany. These observations indicate the value of subtyping YHGs to a highly detailed level in micro geographic regions and add to the existing knowledge based on genome-wide data and mitochondrial data. It is therefore of great use as a reference in forensic casework in the Netherlands in relation to genetic ancestry and geographic origin assessments of suspects or unidentified victims. Moreover, the geographic patterns we observed, in addition to genome wide data, stress the importance of taking geographic origin into account in sampling strategies for control groups and comparing data from subpopulations in epidemiological studies.

The discrepancies between patterns of population substructure of mitochondrial and Y-chromosomal DNA data point to different population histories for women and men in the Netherlands. However, solid explanations for the observed spatial patterns require further multidisciplinary research, including historians and archaeologists and detailed YHG data, similar to what we present here, from past Dutch populations via ancient DNA analysis.

## Supplementary information

supplementary information

supplemental table S4
