# Correction to: The Dutch Y-chromosomal landscape

**DOI:** 10.1038/s41431-019-0528-9

**Published:** 2019-10-23

**Authors:** Eveline Altena, Risha Smeding, Kristiaan J. van der Gaag, Maarten H. D. Larmuseau, Ronny Decorte, Oscar Lao, Manfred Kayser, Thirsa Kraaijenbrink, Peter de Knijff

**Affiliations:** 1 Department of Human Genetics, Leiden University Medical Center, Leiden, The Netherlands; 2 Forensic Biomedical Sciences, Department of Imaging & Pathology, KU Leuven, Leuven, Belgium; 3 Laboratory of Socioecology and Social Evolution, Department of Biology, KU Leuven, Leuven, Belgium; 4 Histories vzw, Mechelen, Belgium; 5 Laboratory of Forensic Genetics and Molecular Archaeology, UZ Leuven, Leuven, Belgium; 6 Department of Genetic Identification, Erasmus MC University Medical Center Rotterdam, Rotterdam, The Netherlands; 7 Present Address: Department of Human Biological Traces, Netherlands Forensic Institute, The Hague, The Netherlands; 8 Present Address: Universitat Pompeu Fabra, Barcelona, Spain; 9 Present Address: CNAG-CRG, Centre for Genomic Regulation (CRG), The Barcelona Institute of Science and Technology, Barcelona, Spain

**Correction to: European Journal of Human Genetics**

10.1038/s41431-019-0496-0

Due to a misunderstanding between the authors, Dr. Lao’s present address was incomplete when the paper was published. The correct present address should read: CNAG-CRG, Centre for Genomic Regulation (CRG), Barcelona Institute of Science and Technology (BIST), Barcelona, and Universitat Pompeu Fabra (UPF), Barcelona, Spain.

