# Supplementary material for: The Dutch Y-chromosomal landscape
Source: Eur J Hum Genet. 2019 Sep 5;28(3):287–99. doi: 10.1038/s41431-019-0496-0 (PMC7029002; doi:10.1038/s41431-019-0496-0)
Supplement: Supplementary file 1 — supplementary information [file 41431_2019_496_MOESM1_ESM.pdf]

SUPPLEMENTARY INFORMATION

The Dutch Y-chromosomal landscape

Eveline Altena, Risha Smeding, Kristiaan van der Gaag, Maarten HD Larmuseau, Ronny Decorte, Oscar Lao, Manfred Kayser, Thirsa Kraaijenbrink, Peter de Knijff

Eur J Hum Genet 2019

Samples

The samples were bought from Sanquin, the only official Dutch blood-collecting organization and exclusively authorized by the Dutch government to sell and or distribute products derived from these donated blood samples. All donors were asked, prior to their donation, if they agreed with the sales of part of their white cells to the Forensic Laboratory for DNA Research (FLDO) of the Leiden University Medical Center for fundamental population genetic research purposes. They were given sufficient time to read an informed consent and informative brochure prior to their donation. Consent was registered by Sanquin, and only the samples from donors who agreed were subsequently sold to the FLDO.

Primer design

A SNaPshot® Multiplex System Kit (Applied Biosystems, Foster City, CA, USA) assay was designed for a core set of 26 SNPs covering all the main YHGs (A-T) and four subgroups of YHG R. Samples assigned to YHG E were further subtyped with a multiplex assay containing 18 SNPs. Samples that were assigned to subgroup E1b-M2 with this multiplex assay were again further subtyped with a multiplex assay containing 21 SNPs. Samples assigned to subgroup R1b were further subtyped with a multiplex assay containing 18 SNPs and one monoplex assay for L23. Further subtyping of samples assigned to YHGs F(xG, H, I/J, K), J and Q was done in monoplex (figure 2). Markers M174 and MEH2 have similar fragments on the X-chromosome and therefore also function as female control.

Primer sequences for M2, M35, M45, M78, M123, M175, M224 and SRY10831 were taken from literature [1]. For the other SNPs reference sequences for each locus were obtained from the BLAST human genome database [2] and PCR-primers were designed for fragments ranging from 66 to 179 bp with Primer3 v.0.2 using default settings [3]. Lengths of designed primers range from 18 to 30 nucleotides. Primers with five or more bases at the 3’ end

complementary to part of another primer were discarded or redesigned to avoid primer-dimer formation and amplicon sequences were checked with BLAST for sequence homology in the human genome.

Primers for minisequencing were designed with the 3' end base corresponding to the last base before the SNP-position using Assay Design Software Version 1.0.6 (Biotage, Uppsala, Sweden). Primers with four or more bases at the 3' end complementary to part of another primer were discarded or redesigned if possible to avoid nonspecific primer-extension and primer lengths were altered by adding a piece of a 'neutral' sequence or a poly-C tail as described by [1]. See table S4 for information on primer and sequencing design.

PCR and sequencing conditions

Each primer pair was validated in a monoplex PCR containing 0.5 ng template DNA from a selection of samples (including a female control sample), 1x PCR buffer containing 1.5mM $MgCl_2$ (Applied Biosystems), 100 µM of each dNTP (GE Healthcare, Little Chalfont, UK) 0.4 µM of each primer (desalted, Biolegio bv, Nijmegen, the Netherlands) and 0.6 units of AmpliTaq Gold® DNA polymerase (Applied Biosystems). The multiplex assays were validated in a PCR with a 12.5 µl reaction volume containing 0.5 ng template DNA, 1x PCR buffer (Applied Biosystems), 6.5mM total $MgCl_2$ (Applied Biosystems), 200 µM of each dNTP and 2.5 units of AmpliTaq Gold® DNA polymerase. Primer concentrations were adjusted to optimize balanced intensity for all markers (see table S4 for primer concentrations). All reactions were performed in a GeneAmp® PCR System 9700 (Applied Biosystems) with a pre-denaturation at 94°C for 10 min, followed by 35 cycles of 30 s at 94°C, 30 s at 60°C, 30 s at 72°C and a final extension for 5 min at 72°C. To eliminate excess primers and dNTPs, 2 µl ExoSAP-IT® (USB®, Affymetrix, Inc, Ohio, USA) was added to the PCR product and incubated at 37°C for 30 min, followed by an incubation at 80°C for 15 min to inactivate enzymes. Minisequencing reactions were performed in a 5 µl reaction volume containing 1 µl purified PCR product, 2.5 µl of SNaPshot multiplex Ready Reaction Mix (Applied Biosystems) and 0.4 µM primer (up to 50 bp HPLC purified, otherwise PAAGE purified, Biolegio bv). Extension primer concentrations were adjusted to optimize balanced intensity for all markers (see table S4 for extension primer concentrations). All reactions were performed with a predenaturation at 96°C for 2 min, followed by 25 cycles of 10 s at 96°C, 5 s at 50°C and 30 s at 60°C. To eliminate unincorporated ddNTPs 1.25 µl SAP® -reagent (USB®) was added and incubated at 37°C for 1 hour. SAP was inactivated by incubation at 75°C for 15 min. 2 µl of the SAP-treated PCR product was analyzed with an

ABI3100 Genetic Analyzer using a 36 cm capillary array, polymer POP4 and Genescan 120 LIZ as internal size standard. Data was analysed using GeneMarker® v1.75 (SoftGenetics). After background subtraction and colour separation, peaks were sorted into bins according to sizes by comparison to the internal size standard. An Excel-sheet was used to transfer exported allele tables and automatically call haplogroups.

Haplogroup prediction

Subgroups of YHG I-M170 were inferred with Whit Athey's Haplogroup Predictor [4,5], based on 16 YSTRs that were previously published for our dataset [6]. Multiple and intermediate alleles were discarded. The prediction was done with the 27 HG analysis mode, using the 37 marker input format, with a minimum haplogroup score of 40 and a minimum probability of 95 %. All three priors (Northwest Europe, East Europe and equal priors) were tested, but there were no discrepancies between the results with the different priors. With the prior Northwest Europe, however, a YHG could be predicted for more samples than with the other priors, therefore the results based on this prior are used.

References

1. Sanchez JJ, Borsting C, Hallenberg C, Buchard A, Hernandez A, Morling N. Multiplex PCR and minisequencing of SNPs--a model with 35 Y chromosome SNPs. Forensic Sci Int. 2003;137:74-84.
2. http://www.ncbi.nlm.nih.gov/blast/
3. http://frodo.wi.mit.edu
4. Athey TW. Haplogroup Prediction from Y-STR Values Using an Allele Frequency Approach. Journal of Genetic Genealogy. 2005;1:1-7.
5. Athey TW. Haplogroup Prediction from Y-STR Values Using a Bayesian-Allele Frequency Approach. Journal of Genetic Genealogy. 2006;2:34-39.
6. Westen AA, Kraaijenbrink T, Clarisse L et al. Analysis of 36 Y-STR marker units including a concordance study among 2085 Dutch males. Forensic Sci Int Genet. 2015;14:174-81.

Supplementary figures

Figure S1. Plot of the first two dimensions from the correspondence analysis with outliers.

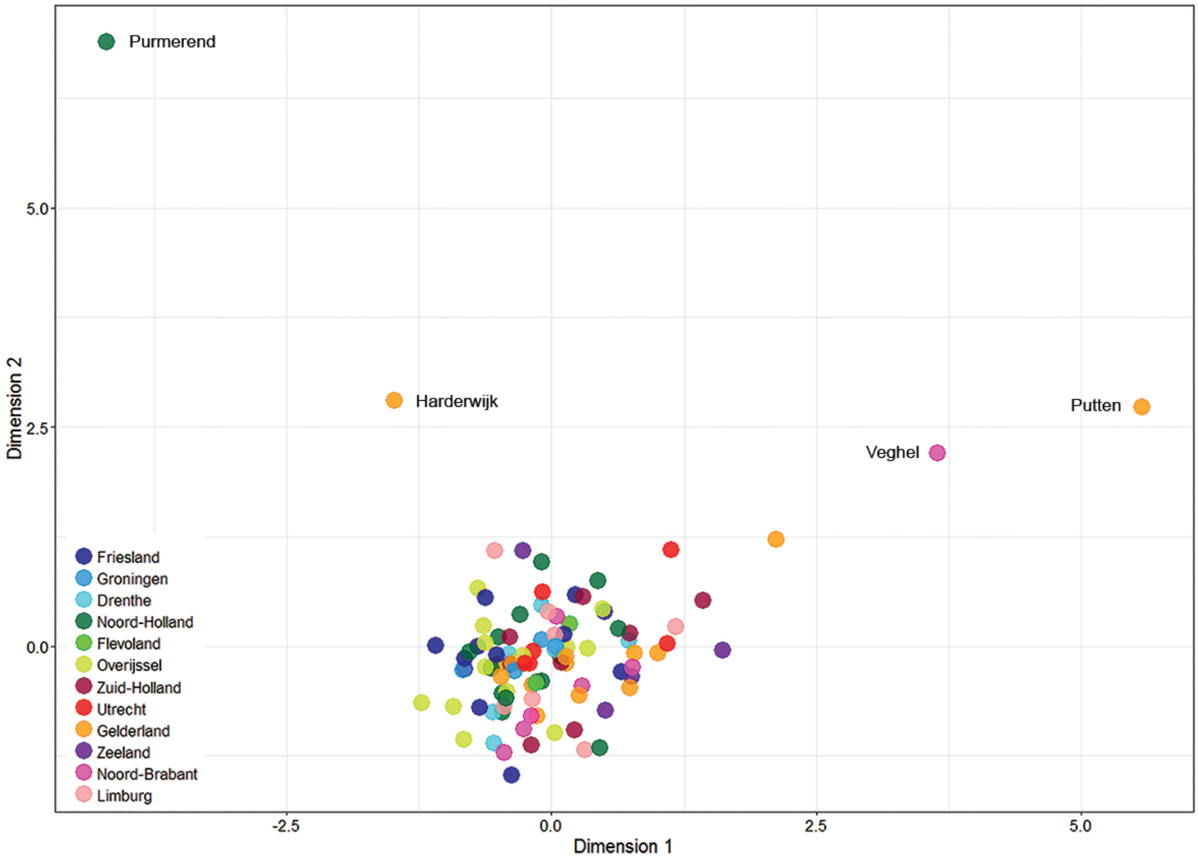

Figure S2. Moran's I spatial autocorrelograms of YHGs with a significant overall *p*-value ($p \leq 0.5$).

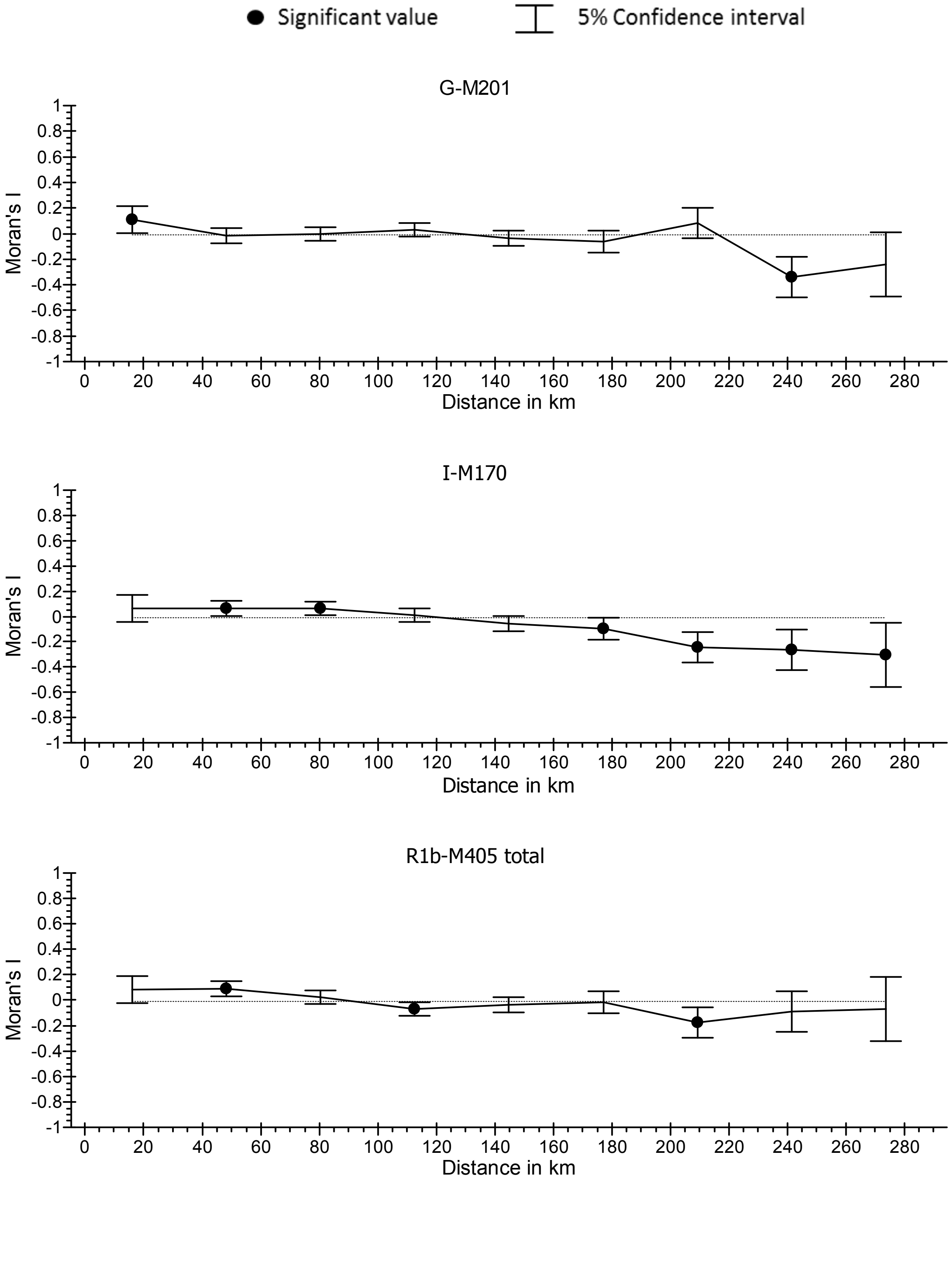

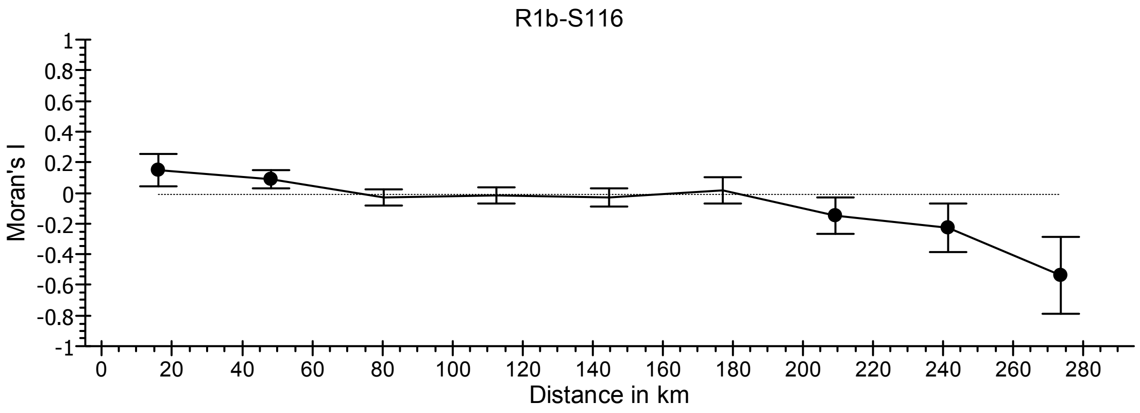

R1b-S116
Moran's I
Distance in km

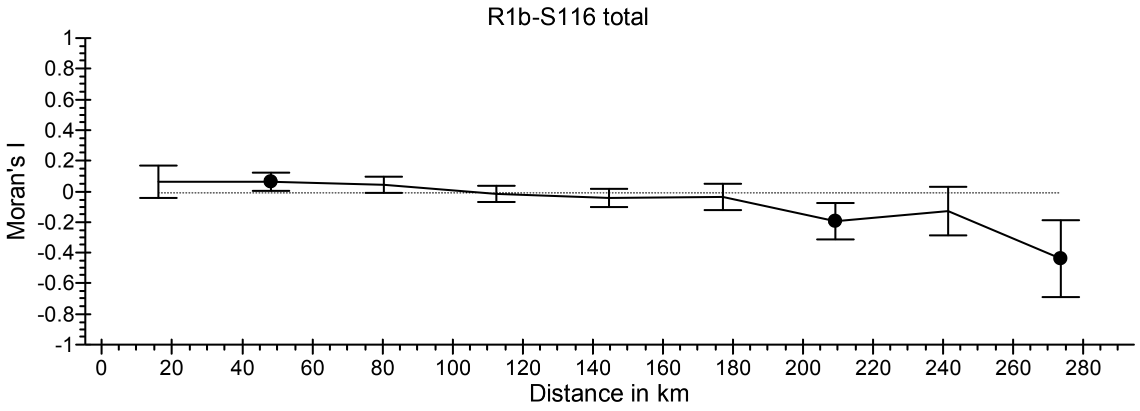

R1b-S116 total
Moran's I
Distance in km

Figure S3. Prediction surface maps of YHGs with a proportion of ≥1 % in the Dutch dataset in phylogenetic order.

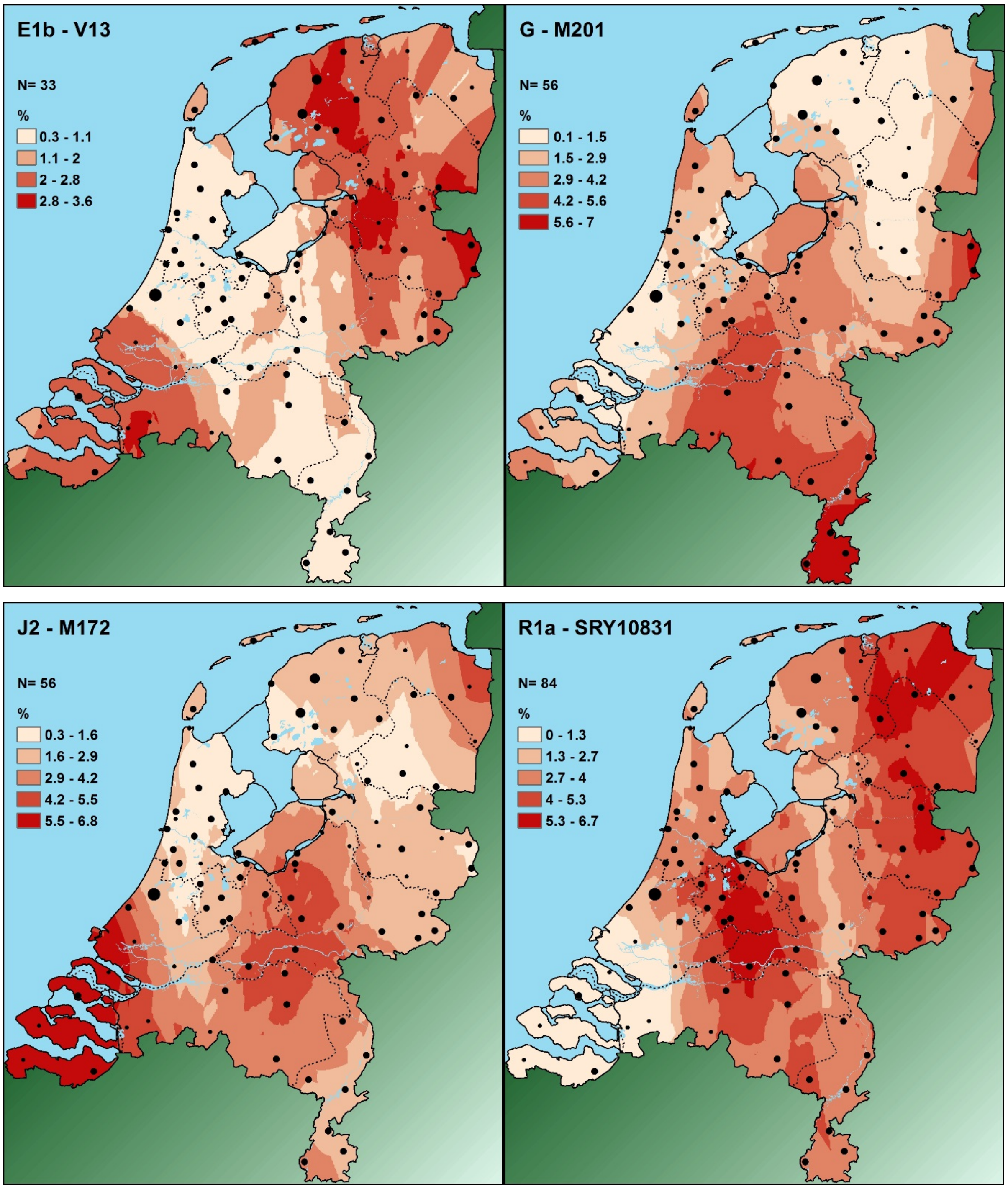

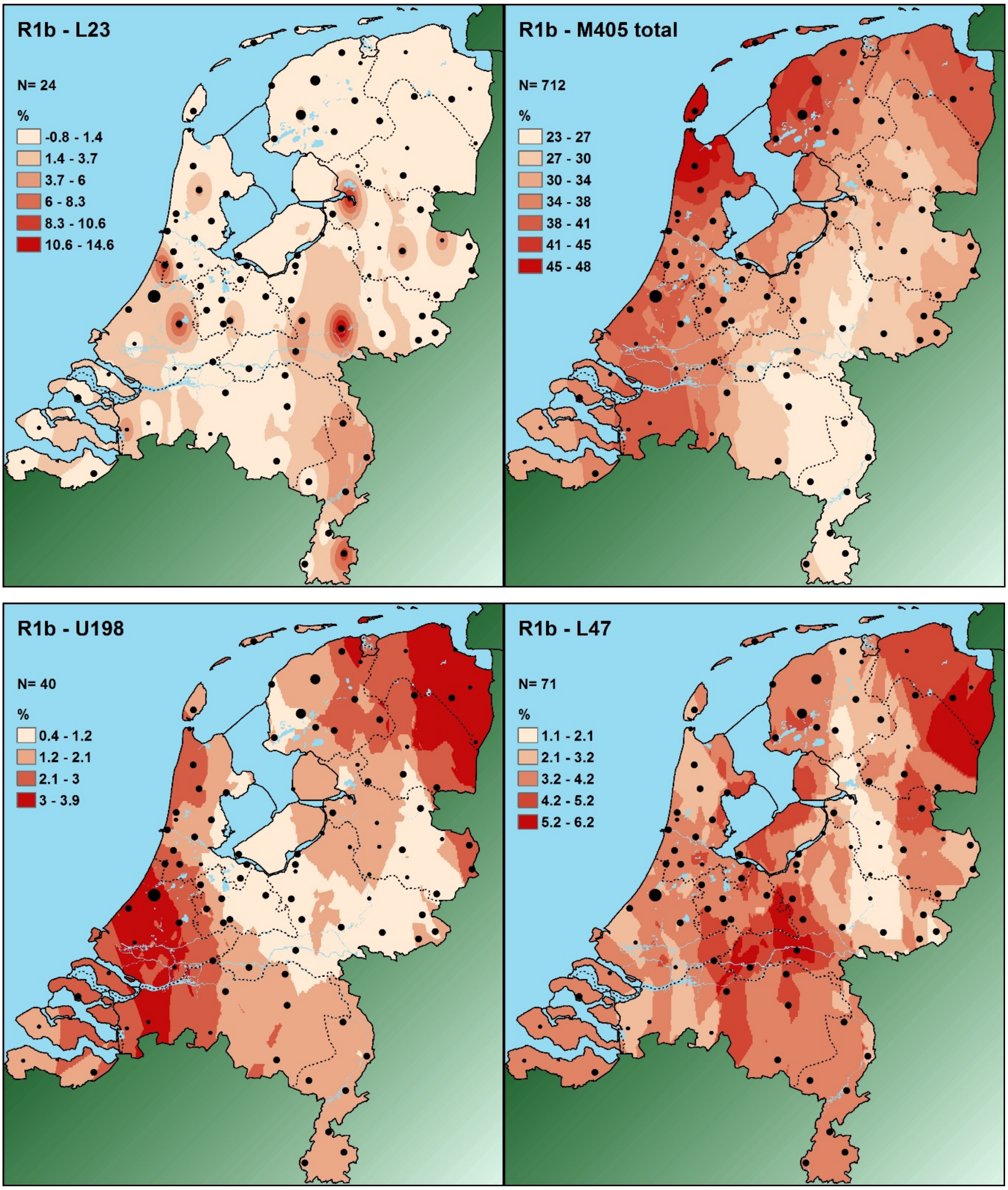
R1b - L23
N= 24
%
-0.8 - 1.4
1.4 - 3.7
3.7 - 6
6 - 8.3
8.3 - 10.6
10.6 - 14.6
R1b - M405 total
N= 712
%
23 - 27
27 - 30
30 - 34
34 - 38
38 - 41
41 - 45
45 - 48
R1b - U198
N= 40
%
0.4 - 1.2
1.2 - 2.1
2.1 - 3
3 - 3.9
R1b - L47
N= 71
%
1.1 - 2.1
2.1 - 3.2
3.2 - 4.2
4.2 - 5.2
5.2 - 6.2

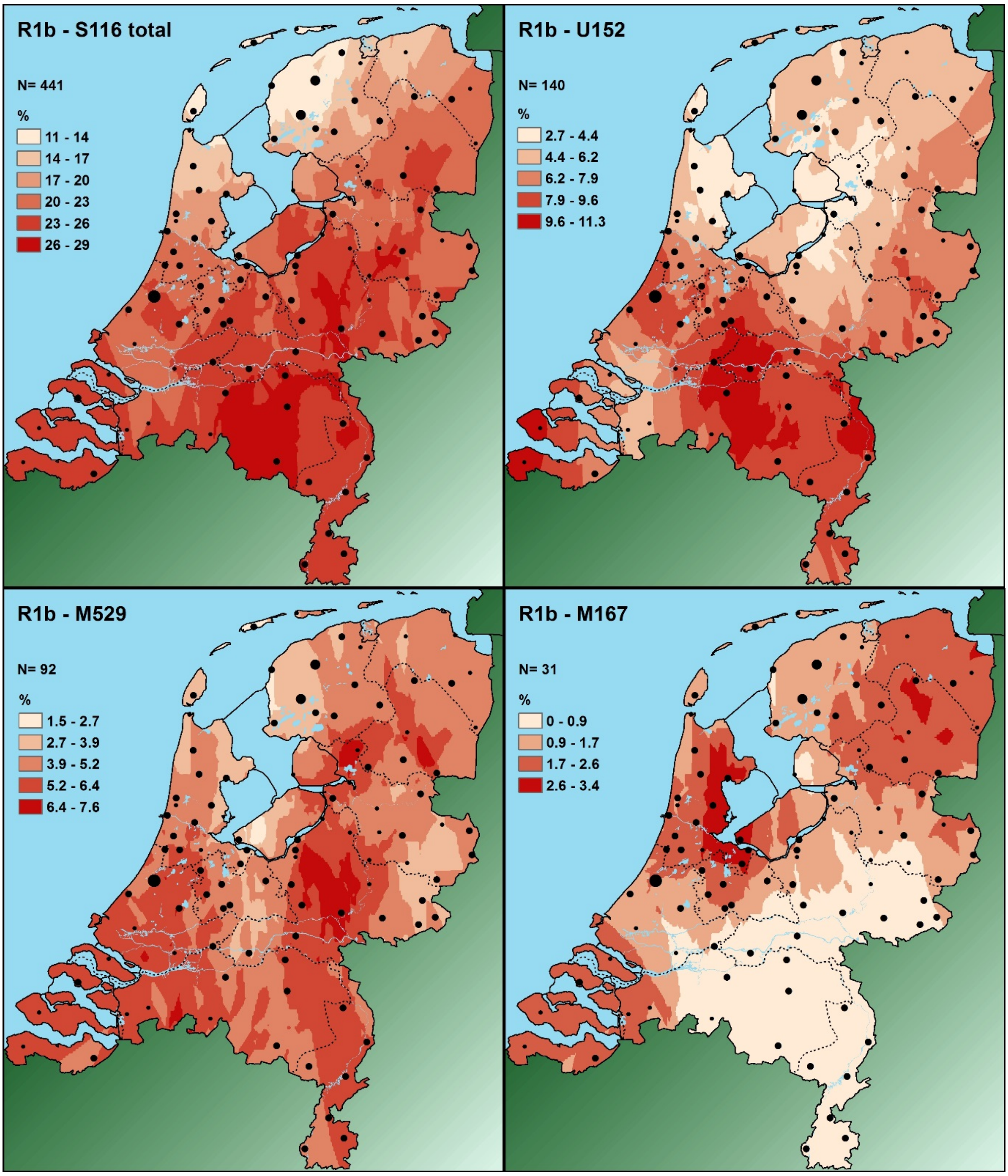
R1b - S116 total
N= 441
%
11 - 14
14 - 17
17 - 20
20 - 23
23 - 26
26 - 29
R1b - U152
N= 140
%
2.7 - 4.4
4.4 - 6.2
6.2 - 7.9
7.9 - 9.6
9.6 - 11.3
R1b - M529
N= 92
%
1.5 - 2.7
2.7 - 3.9
3.9 - 5.2
5.2 - 6.4
6.4 - 7.6
R1b - M167
N= 31
%
0 - 0.9
0.9 - 1.7
1.7 - 2.6
2.6 - 3.4

Supplementary tables

Table S1. Locations, provinces, coordinates and sample size per location. When locations are pooled, the coordinates from the underlined location are used.

Province	Location	Latitude	Longitude	N
Friesland	Dokkum	53.3333	6	23
N=298	Drachten	53.1	6.1	20
	Harlingen	53.1756	5.43	27
	Heerenveen	52.95	5.9333	21
	Hollum/Nes	53.45	5.6333	16
	Joure	52.9667	5.7833	23
	Kollum	53.2833	6.15	11
	Koudum	52.9167	5.45	24
	Leeuwarden	53.2	5.7833	40
	Midsland	53.3833	5.2833	22
	Oosterwolde	53	6.3	22
	Sneek	53.0333	5.6667	49
Groningen	Delfzijl	53.3333	6.9167	14
N=62	Hoogezand/Veendam	53.1	6.8833	20
	Winschoten	53.15	7.0333	15
	Winsum/Zuidhorn	53.3333	6.5167	13
Drenthe	Assen/Eelde	53.1167	6.5833	24
N=131	Beilen/Borger	52.8667	6.5167	16
	Coevorden	52.6667	6.75	21
	Emmen/Ter Apel	52.8833	7.0667	16
	Hoogeveen	52.7333	6.4833	29
	Meppel	52.7	6.2	25
Noord-Holland	Amstelveen	52.3	4.8667	19
N=297	Castricum	52.55	4.6667	20
	Den Burg	53.05	4.8	20
	Den Helder	52.956	4.7667	19
	Haarlem	52.3667	4.65	20
	Heemskerk	52.5167	4.6667	18
	Heerhugowaard	52.6667	4.85	21
	Hilversum	52.2333	5.1833	20
	Hoofddorp	52.3	4.7	20
	Hoorn	52.65	5.0667	20
	Huizen	52.3	5.2333	20
	IJmuiden	52.4667	4.6	20
	Purmerend	52.5167	4.95	20
	Schagen	52.7833	4.8	20
	Zaanstad	52.4333	4.8167	20

Continuation of Table S1.

Province	Location	Latitude	Longitude	N
Flevoland	Almere	52.35	5.1667	20
N=37	Urk	52.6667	5.6	17
Overijssel	Dalfsen	52.5	6.2667	17
N=250	Denekamp	52.3833	7	24
	Deventer	52.25	6.2	17
	Genemuiden	52.625	6.05	16
	Haaksbergen	52.15	6.7333	22
	Hardenberg	52.5667	6.6167	24
	Kampen	52.55	5.9167	25
	Losser	52.2667	7.0167	20
	Markelo	52.2333	6.5	17
	Nijverdal	52.3667	6.4667	20
	Raalte	52.3833	6.2833	19
	Steenwijk	52.7833	6.1167	11
	Tubbergen	52.4167	6.7833	18
Zuid-Holland	Den Haag	52.083	4.3	28
N=236	Dordrecht	51.8	4.667	17
	Gorinchem	51.833	4.967	20
	Gouda	52.016	4.7	20
	Hillegom	52.3	4.5833	20
	Leiden	52.15	4.5	96
	Sommelsdijk	51.767	4.15	18
	Vlaardingen	51.917	4.35	17
Utrecht	Amersfoort	52.15	5.3833	20
N=122	IJsselstein	52.0167	5.05	20
	Maarssenbroek	52.1333	5.0333	20
	Mijdrecht	52.2	4.8667	20
	Nieuwegein	52.0333	5.1	21
	Woerden	52.0833	4.9167	21

Continuation of Table S1.

Province	Location	Latitude	Longitude	N
Gelderland	Aalten	51.9333	6.5833	23
N=297	Barneveld	52.1333	5.5833	20
	Doetinchem	51.967	6.299	20
	Druten	51.8833	5.6167	20
	Ede	52.033	5.667	20
	Elburg	52.45	5.8333	19
	Ermelo	52.3	5.6167	20
	Groenlo	52.05	6.6167	23
	Harderwijk	52.35	5.6333	21
	Heerde	52.3833	6.05	19
	Putten	52.2667	5.6167	10
	Velp	51.994	5.977	20
	Winterswijk	51.9667	6.7167	23
	Zaltbommel	51.8	5.25	20
	Zutphen	52.1333	6.2	19
Zeeland	Hulst	51.283	4.05	21
N=78	Middelburg	51.5	3.617	19
	Oostburg	51.333	3.5	18
	Zierikzee	51.65	3.916	20
Noord-Brabant	Alphen	51.4833	4.95	19
N=135	Bergen op Zoom	51.5	4.3	18
	Oss	51.767	5.534	20
	Roosendaal	51.533	4.467	18
	Valkenswaard	51.35	5.4667	20
	Veghel	51.6167	5.55	20
	Waalwijk	51.6833	5.0667	20
Limburg	Heerlen	50.9	5.9833	20
N=142	Maastricht	50.85	5.6833	20
	Roermond	51.2	6	22
	Sittard	51	5.867	20
	Venlo	51.3667	6.1667	20
	Venray	51.533	5.984	20
	Weert	51.25	5.7167	20

Table S2. Synchronization of YHGs between the Dutch and Flanders datasets for the combined dataset. In grey YHGs that were pooled to come to a consensus YHG. L11=P310, M405=U106, S116=P312, M167=SRY2627.

**Dutch dataset YHG**	**Combined dataset Consensus YHG**	**Flanders dataset YHG**
A	**A (xBT)**	Y(xBT)
E1b - U290	**E1b - U290**	E - U290
E1b - M215	**E1b - M215**	E - M215*
E1b - M35	**E1b - M35**	E - M35
E1b - M78	**E1b - M78**	E - M78*
E1b - V12	**E1b - V12**	E - V12*
E1b - V13	**E1b - V13**	E - V13*
E1b - V22	**E1b - V22**	E - V22*
E1b - M81	**E1b - M81**	E - M81*
E1b - M123	**E1b - M123**	E - M123*
		E - M34*
F3 - P96	**F3 - P96**	-
G - M201	**G - M201**	G - M406*
		G - P15*
		G - U1*
		G - U8*
H1 - M69	**H1 - M69**	-
I - M170	**I - M170**	I - M253*
		I - P109
		I - P215*
		I - P37.2*
		I - M223*
		I - M284
		I - P78
		I - P95
J - M304	**J - M304 (xM172)**	J - M267*
		J - P58*
J2 - M172	**J2 - M172**	J - M410*
		J - M67*
		J - M92*
		J - M319
		J - M241*

**Dutch dataset YHG**	**Combined dataset Consensus YHG**	**Flanders dataset YHG**
L - M20	**L - M20**	L-M27*
		L - M317*
N - M231	**N - M231**	-
O - M175	**O - M175**	-
Q1a - MEH2	**Q1 - P36.2**	Q - P36.2*
R1 - M173	**R1 - M173**	R - M173*
R1a - SRY10831	**R1a - SRY10831**	R - SRY10831.2*
		R - M198*
R1b - M343	**R1b - M343**	R - M343
		R - P25*
R1b - P297	**R1b - P297**	R - P297*
R1b - M269	**R1b - M269**	R - M269*
R1b - L23		R - P310*
R1b - M412		
R1b - L11		
R1b - M405	**R1b - M405**	R - U106*
		R - Z18
		R - Z381*
R1b - U198	**R1b - U198**	R - U198
R1b - L48	**R1b - L48**	R - L48
R1b - L47		
R1b - S116	**R1b - S116**	R - P312*
		R - Z195
R1b - U152	**R1b - U152**	R - U152*
		R - L2*
		R - L20
R1b - M529	**R1b - M529**	R - M529*
R1b - M167	**R1b - M167**	R - SRY2627
T - M70	**T - M70**	T - P77
		T - L208*
		T - L131*

Table S3. YHG proportions in the Dutch dataset for predicted subgroups of YHG I-M170. YHGs with a proportion of ≥1 % are marked in grey.

YHG I Predicted	#	%
I1	389	18.66
I2a (xI2a1)	18	0.86
I2a1	2	0.10
I2b (xI2b1)	16	0.77
I2b1	102	4.89
G2a	4	0.19
O3	1	0.05
Unpredictable	48	2.30
**Total**	**580**	**27.82**
